# Intersection of Aging and Particulate Matter 2.5 Exposure in Real World: Effects on Inflammation and Endocrine Axis Activities in Rats

**DOI:** 10.1155/2024/8501696

**Published:** 2024-06-27

**Authors:** Cuiying Liu, Jian Yang, Longfei Guan, Liwei Jing, Shuqin Xiao, Liu Sun, Baohui Xu, Heng Zhao

**Affiliations:** ^1^School of Nursing, Capital Medical University, Beijing, China; ^2^China-America Institute of Neuroscience, Beijing Luhe Hospital, Capital Medical University, Beijing, China; ^3^Department of Surgery, Stanford University School of Medicine, Stanford, CA 94305, USA; ^4^Beijing Institute of Brain Disorders, Capital Medical University, Beijing, China

## Abstract

Exposure to particulate matter 2.5 (PM2.5) is detrimental to multiple organ systems. Given the factor that aging also alters the cellularity and response of immune system and dysfunction of hypothalamic-pituitary-adrenal, -gonad and -thyroid axes, it is imperative to investigate whether chronic exposure to PM2.5 interacts with aging in these aspects. In this study, two-months-old Sprague–Dawley rats were exposed to real world PM2.5 for 16 months. PM2.5 exposure diminished the relative numbers of CD4^+^ T cells and CD8^+^ T cells and increased the relative number of B cells in the peripheral blood of male rats. Conversely, only reduced relative number of CD4^+^ T cells was seen in the blood of female rats. These shifts resulted in elevated levels of proinflammatory factors interleukin-6 and tumor necrosis factor-*α* in the circulatory systems of both sex, with females also evidencing a rise in interleukin-1*β* levels. Moreover, heightened interleukin-6 was solely discernible in the hippocampus of female subjects, while increased tumor necrosis factor-*α* concentrations were widespread in female brain regions but confined to the male hypothalamus. Notable hormonal decreases were observed following PM2.5 exposure in both sex. These comprised declines in biomolecules such as corticotrophin-releasing hormone and cortisol, generated by the hypothalamic-pituitary-adrenal axis, and thyroid-releasing hormone and triiodothyronine, produced by the hypothalamic-pituitary-thyroid axis. Hormonal elements such as gonadotropin-releasing hormone, luteinizing hormone, and follicle-stimulating hormone, derived from the hypothalamic-pituitary-gonad axis, were also diminished. Exclusive to male rats was a reduction in adrenocorticotropic hormone levels, whereas a fall in thyroid-stimulating hormone was unique to female rats. Decreases in sex-specific hormones, including testosterone, estradiol, and progesterone, were also noted. These findings significantly enrich our comprehension of the potential long-term health repercussions associated with PM2.5 interaction particularly among the aging populace.

## 1. Introduction

Inhaling particulate matter 2.5 (PM2.5), the particles with a diameter of less than 2.5 micrometers, has severe health impact [1]. These particles emanate from various sources such as including industrial activities, transportation, and wildfires enter lungs and circulate in the bloodstream and contribute to the pathogenesis of respiratory, cardiovascular, cerebral vascular, and other organ systems [1–3]. Although having a great public health impact, the precise mechanisms by which PM2.5 is involved in multiple organ disorders are not fully understood.

PM2.5 exposure has been reported to influence immune function, provoke inflammatory reactions, and alter the activities of endocrine systems including the hypothalamic-pituitary-adrenal (HPA), hypothalamic-pituitary-gonad (HPG), and hypothalamic-pituitary-thyroid (HPT) axes [4–8]. These inflammatory reactions may interact with the endocrine systems and further modulate systemic inflammation. Yet, conflicting findings remain in published studies regarding the influence of PM2.5 exposure on these axes. Alternations in hormone levels are influenced by several factors, including personal susceptibility, natures of environmental pollutants, and exposure duration [9]. While PM2.5 was reportedly associated with either elevated or reduced levels for HPA axis hormones [6, 10], the reported influence was inconsistent and varied by pollutants [11–16].

In our previous studies, the exposure to PM2.5 for 6 months led to systemic inflammation and disrupted endocrine function in adult rats, as evidenced by increased levels of systemic inflammatory markers in the peripheral blood, brain, and endocrine glands [5]. It also diminished hormone levels secreted by both HPT and HPG axes and escalated inflammatory responses in thyroid and gonads [6].

Aging can modify individual susceptibility and responses to environmental stressors including PM 2.5 [17]. Aging not only attenuates immunological function but also creates chronic low-level inflammation status known as inflammaging [18–20]. In addition, aging is also associated with disfunctions of HPA, HPT, and HPG that potentially amplifies the host responses to stress, metabolism, and reproduction [4, 21–24]. Hence, it warrants investigating the cumulative effects of chronic PM2.5 exposure and aging on these systems [10, 25–27]. However, most previous studies have focused on short term, and we still lacked the studies on the interplay between long-term PM2.5 exposure and aging on immune and endocrine system activity.

The understanding of the interplay of aging and PM2.5 in endocrine and immune systems has significant public health interests. It may also guide developing effective strategies for mitigating adverse health outcomes from air pollution, particularly in vulnerable individuals, and inform policy and healthcare decisions in managing PM2.5 exposure.

This study used a real-world PM2.5 exposure system and investigated the influence of long-term PM2.5 exposure on the immune and neuroendocrine systems in rats. By exposing 2-months-old rats to PM2.5 for 16 months, we comprehensively evaluated regional and systemic inflammation and neuroendocrine secretion function to provide some insights into the adverse effects of chronic PM2.5 exposure in aging population.

## 2. Materials and Methods

### 2.1. Rats

Two-months-old specific pathogen free male and female Sprague–Dawley rats were purchased from Vital River Laboratory Animal Technology Co., Ltd, Beijing, China. Following 3 day adaptation in temperature-controlled rooms with 12-hour light/dark cycles, animals were housed in climate-controlled chambers with a 12-hour light/dark cycle, unlimited access to sustenance and hydration and a relative humidity of 50%–60%, and randomized to exposed to filtered air (FA) and unfiltered air (PM2.5 exposure). Experimental protocols complied with the guidelines endorsed by the Animal Care and Use Committee of Capital Medical University.

### 2.2. Long-Term PM2.5 Exposure

Rats underwent PM2.5 exposure for 16 months starting on November 14, 2017, and ending on March 14, 2019, in a PM2.5 exposure apparatus with 3 rats in each enclosure previously described [5, 6, 28]. To maintain filtered air condition, a specialized air filter was positioned at the inlet to remove PM2.5 particles. Daily ambient PM2.5 concentrations were monitored during the whole exposure period via a pDR1500 particulate meter from Thermo Fisher Scientific (Waltham, MA, USA). No mortality was noted for the 16-month exposure to PM2.5. At the end of experiments, all rats were euthanized and cortex, hippocampus, hypothalamus, and peripheral blood were harvested.

### 2.3. qRT-PCR Assay

Total RNA was extracted from cortex, hippocampus, and hypothalamus utilizing TRNzol reagent (Invitrogen, Carlsbad, CA) according to the manufacturer's guidelines. cDNA was synthesized via the PrimeScript™ RT reagent kit with gDNA Eraser from TaKaRa Bio, China. Real-time PCR amplification was conducted on the Prism 7500 real-time PCR instrument (Applied Biosystems, CA, USA) using gene-specific primer sets including housekeeping gene glyceraldehyde 3-phosphate dehydrogenase (GAPDH) (Table 1).

### 2.4. Flow Cytometric Analysis

Heparinized peripheral blood (0.2–0.5 mL) was collected from retroorbital vein of anesthetized rats. Leukocytes were isolated using Ficoll gradient centrifugation method as described previously [5]. CD4^+^ T cells (CD3^+^CD4^+^), CD8^+^ T cells (CD3^+^CD8^+^), and B cells (CD45RA ^+^ CD3^−^) were stained by different antibodies purchased from BD Pharmingen ™ (San Jose, CA, USA). The data were analyzed using a flow cytometer BD LSRII and BD FACSDiva ™ Software (Version6.1.3).

### 2.5. ELISA Assays

Serum levels for inflammatory cytokines and hormones were determined using commercial ELISA kits from Shanghai Sixin Biotechnology Co., Ltd, Shanghai, China. Cytokines were interleukin (IL)-6, IL-1*β*, and tumor necrosis factor (TNF)-*α*. Hormones were corticotrophin-releasing hormone (CRH), adrenocorticotropic hormone (ACTH), and cortisol (CORT), thyroid-releasing hormone (TRH), thyroid-stimulating hormone (TSH), triiodothyronine (T3), and thyroxine (T4), gonadotropin-releasing hormone (GnRH), luteinizing hormone (LH), follicle-stimulating hormone (FSH), testosterone (T), estradiol (E2), and progesterone (PROG).

### 2.6. Statistical Analysis

GraphPad Prism (Ver 8.0.2, GraphPad Software, ILLC, San Diego, CA, USA) was used for statistical analyses. All data were normally distributed as tested by the Shapiro–Wilk normality test and presented as the mean and the standard error of the mean (SEM). Student's *T*-test or analysis of variance (ANOVA, one way, two way, or three way) followed by the Bonferroni post hoc test was used to determine the statistical significance. A *P* value below 0.05 was considered statistically significant.

## 3. Results

### 3.1. Monthly Enclosure PM2.5 Levels and Rat Body Weight Gain during the 16-Month PM2.5 Exposure

**Figure 1:**
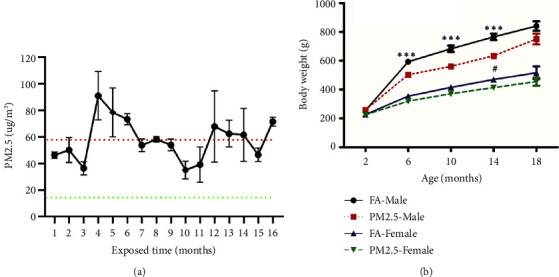
Ambient air PM2.5 levels and rat body weight during the 16-month exposure. Monthly mean PM2.5 concentrations during the 16-month exposure period (a). PM2.5 concentrations were measured using an individual particle monitor. Rat body weights were recorded every four months during the 16-month exposure period, beginning at two months of age. Red dotted line presents the average PM2.5 concentration during the exposure period, while green dotted line presents the annual average PM2.5 National Ambient Air Quality Standard in China. (b) Body weights in male and female rats exposed to unfiltered (PM2.5) and filtered air (FA). ^*∗∗∗*^*P* < 0.001 as compared to male rats exposed to PM2.5. ^#^*P* < 0.05 as compared to female rats exposed to PM2.5 significant, *n* = 6–9 rats in each group.

Monthly average PM2.5 concentrations in exposure chambers during the 16-month exposure period was presented Figure 1(a). Average daily PM2.5 concentration was 57.9 *μ*g/m^3^ in unfiltered chambers, which was nearly four folds of annual average PM2.5 levels recommended by China National Ambient Air Quality Standard (15 *μ*g/m^3^) and two folds of daily PM2.5 level recommended the World Health Organization Air Quality Guidelines (25 *μ*g/m^3^). However, tPM2.5 concentration in FA chambers was 3.5 *μ*g/m^3^. As expected, body weight increased with increasing age in both male and female rats regardless of whether they were exposed to filtered or unfiltered air. In male rats, PM2.5 exposure led to a significant reduction in body weight gain at the age of 6, 10, and 14 months as compared to filtered air exposure. However, this effect on body weight was only noted in female rats at the age of 14 months (Figure 1(b)).

### 3.2. Long-Term PM2.5 Exposure Alters the Relative Number of Peripheral Blood Lymphocytes in Rats

**Figure 2:**
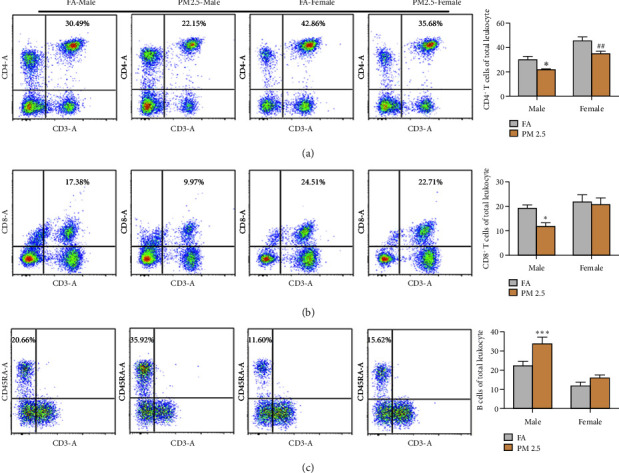
Impact of 16-month PM2.5 exposure on peripheral blood lymphocytes. Flow cytometry analysis revealed significant reductions in the proportions of CD4^+^ T cells (CD3^+^CD4^+^) and CD8^+^ T cells (CD3^+^CD8^+^) and increases in the percentage of B cells (CD45RA^+^CD3^−^) in male rats. Conversely, female rats only showed a reduction in the percentage of CD4^+^ T cells. *n* = 4 rats in each group, ^*∗*^*P* < 0.05 and ^*∗∗∗*^*P* < 0.001 for male rats, and ^##^*P* < 0.01 for female rats compared to filtered air (FA) exposure. PM2.5: particulate matter 2.5. (a) Analysis of the proportion of CD4^+^ T cells in the blood. (b) Analysis of the proportion of CD8^+^ T cells in the blood. (c) Analysis of the proportion of B cells in the blood.

To assess the impact of long-term PM2.5 exposure on peripheral lymphocytes, we stained peripheral blood mononuclear cells with differentially fluorochrome-conjugated mAbs against CD3, CD4, CD8, and CD45RA (Figure 2). In male rats, PM2.5 exposure resulted in decreases in the percentages of CD4^+^ T cells (CD3^+^CD4^+^) and CD8 T cells (CD3^+^CD8^+^) and an increase in the percentage of B cells (CD45RA^+^CD3^−^) in the peripheral blood as compared to filtered air exposure. Conversely, PM2.5 exposure reduced the percentage of CD4^+^ T cells without affecting on CD8^+^ T cells and B cells as compared to filtered air exposure in female rats.

### 3.3. Long-Term PM2.5 Exposure Modulates Systemic Levels of Inflammatory Cytokines

**Figure 3:**
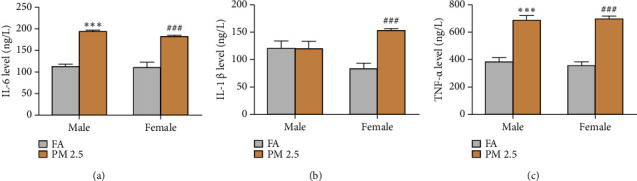
Effect of 16-month PM2.5 exposure on serum inflammatory cytokine levels. Male and female rats exposed to PM2.5 exhibited significant elevations in the levels of IL-6, IL-1*β*, and TNF-*α* as compared to filtered air (FA) exposure. *n* = 4 rats in each group, ^*∗∗∗*^*P* < 0.001 for male rats, and ^###^*P* < 0.001 for female rats as compared to filtered air (FA). PM2.5: particulate matter 2.5. (a) IL-6 level in the blood. (b) IL-1*β* level in the blood. (c) TNF-*α* level in the blood.

To evaluate whether long-term PM2.5 exposure promotes systemic inflammation, the serum levels of 3 proinflammatory cytokines (IL-1*β*, IL-6 and TNF-*α*) were measured by ELISA (Figure 3). Both IL-6 and TNF-*α* levels were significantly higher in male and female mice exposed to PM2.5 than those to filtered air. However, increase in serum IL-1*β* levels were only noted for female mice between two exposure groups.

### 3.4. Long-Term PM 2.5 Exposure Alters Proinflammatory Cytokine Gene Expression in Cortex, Hippocampus, and Hypothalamus

**Figure 4:**
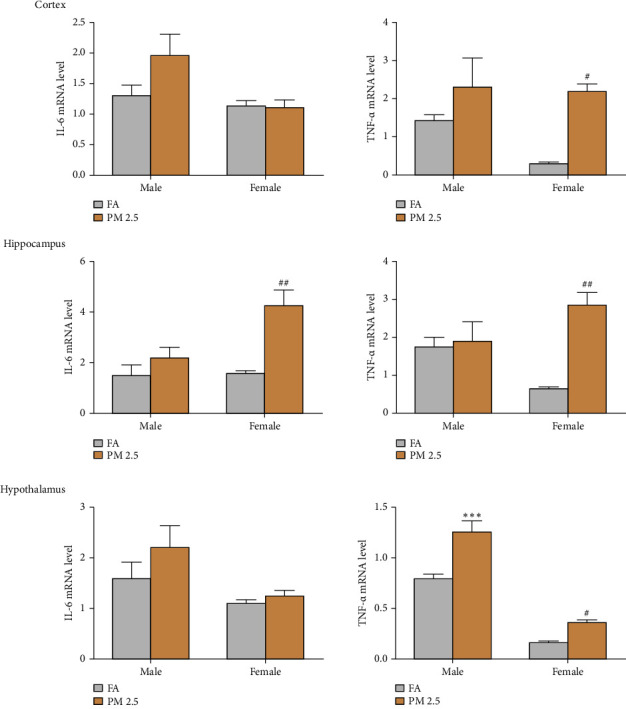
Impact of 16-month PM2.5 exposure on the mRNA expression levels of inflammatory cytokines in the cortex, hippocampus, and hypothalamus from male and female rats. The exposure to PM2.5 significantly upregulated the mRNA expression levels of these cytokines in the cortex, hippocampus, and hypothalamus of male and female rats. *n* = 4 rats in each group, ^*∗∗∗*^*P* < 0.001 for male rats, and ^#^*P* < 0.05 and ^##^*P* < 0.01 for female rats as compared to filtered air (FA) exposure. PM2.5: particulate matter 2.5.

Next, to evaluate the inflammation in cortex, hippocampus, and hypothalamus, we assayed mRNA levels for IL-6 and TNF-*α* in these three tissues via real-time quantitative RT-PCR (Figure 4). The influence on both cytokine mRNAs varied in cytokines and tissues. In cortex, though PM2.5 exposure increased IL-6 mRNA levels in male rats as compared to filtered air exposure, but there was no significant difference between two groups. While TNF-*α* mRNA levels were higher in PM2.5 exposure that those in filtered air exposure in both male and females, the significance was only seen in female rats between two exposure groups. In hippocampus, PM2.5 exposure significantly increased both IL-6 and TNF-*α* mRNAs in female, but not male rats, as compared to filtered air exposure. In hypothalamus, PM2.5 exposure dramatically increased TNF-*α*, but not IL-6, mRNA levels in both male and female rats.

### 3.5. Long-Term PM 2.5 Exposure Alters Serum Hormone Levels in Rats

**Figure 5:**
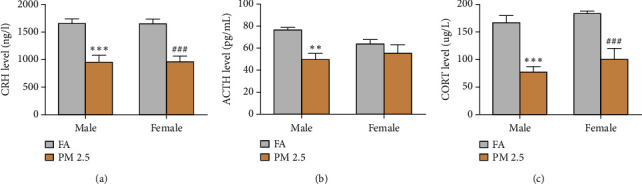
Effect of 16-month PM2.5 exposure on serum hormones levels of hypothalamic-pituitary-adrenal axis in male and female rats. PM2.5 exposure significantly reduced the levels of these hormones in male and female rats. *n* = 6–8 rats in each group, ^*∗∗*^*P* < 0.01, ^*∗∗∗*^*P* < 0.001 for male rats, and ^###^*P* < 0.001 for female rats as compared to filtered air (FA) exposure. CRH: corticotrophin-releasing hormone; ACTH: adrenocorticotropic hormone; and CORT: cortisol. (a) CRH level in the blood. (b) ACTH level in the blood. (c) CORT level in the blood.

**Figure 6:**
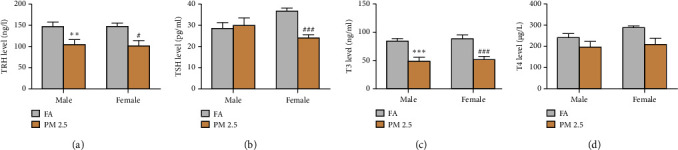
Effect of 16-month PM2.5 exposure on the serum hormone levels of hypothalamic-pituitary-thyroid axis in male and female rats. PM2.5 significantly decreased serum levels of TRH and T3 in male and female rats and reduced serum TSH levels in female rats. *n* = 6–8 rats in each groups, ^*∗∗*^*P* < 0.01 and ^*∗∗∗*^*P* < 0.001 for male rats, and ^#^*P* < 0.05 and ^###^*P* < 0.001 for female rats as compared to filtered air (FA) exposure. TRH: thyroid-releasing hormone; TSH: thyroid-stimulating hormone; T3: triiodothyronine (T3); T4: thyroxine (T4); and PM2.5: particulate matter 2.5. (a) TRH level in the blood. (b) TSH level in the blood. (c) T3 level in the blood. (d) T4 level in the blood.

**Figure 7:**
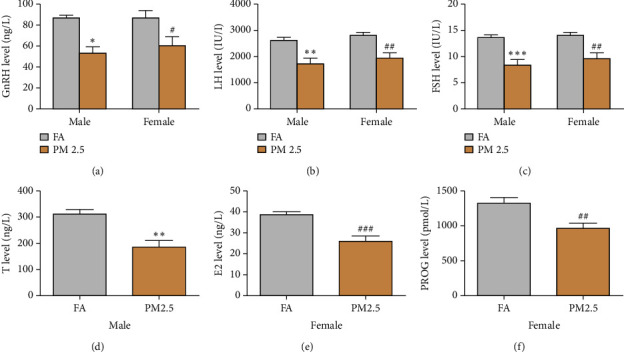
Effect of 16-month PM2.5 exposure on the serum hormone levels of hypothalamic-pituitary-gonad axis in male and female rats. PM2.5 exposure significantly decreased the levels of all hormones, except for T4, in male and female rats. T levels were reduced in male rats, and E2 and PROG levels were reduced in female rats. *n* = 6–8 rats in each group, ^*∗*^*P* < 0.05 and ^*∗∗*^*P* < 0.01, ^*∗∗∗*^*P* < 0.001 for male rats, and ^#^*P* < 0.05, ^##^*P* < 0.01, and ^###^*P* < 0.001 for female rats as compared to filtered air (FA) exposure. GnRH: gonadotropin-releasing hormone, LH: luteinizing hormone; FSH: follicle-stimulating hormone; T: testosterone; E2: estradiol; and PROG: progesterone. (a) GnRH level in the blood. (b) LH level in the blood. (c) FSH level in the blood. (d) T level in the blood. (e) E2 level in the blood. (f) PROG level in the blood.

Finally, we measured various hormone levels in rats exposed to PM2.5 and filtered air for 16 months. In general, PM2.5 exposure reduced serum hormone levels as compared to filtered air exposure (Figures 5–7). PM2.5 exposure led to significant decreases in the levels of CRH and CORT in both male and female rats, but only a reduction in serum ACTH levels rats was noted in male as compared to filtered air exposure (Figure 5), As seen in Figure 6, the serum levels of TRH and T3 were significantly decreased in both male and female rats following long-term PM2.5 exposure as compared to filtered air exposure. Significantly reduced TSH levels were only observed in female, but not male, rats as compared to filtered air exposure. However, PM2.5 exposure had no impact on serum levels of T4 in either male or female rats.

Regarding serum hormone levels secreted from HPG axis (Figure 7), PM2.5 exposure significantly reduced the levels of GnRH, LH, and FSH in both male and female rats as compared to filtered air exposure. Similarly, PM2.5 exposure also significantly reduced *T* levels in male rats and the levels of E2 and PROG in female rats as compared to filtered air exposure.

## 4. Discussion

In this study, we scrutinized the impact of long-term PM2.5 exposure on the well-being of both male and female rats during their aging trajectory. Utilizing an authentic PM2.5 exposure apparatus in a real-world setting, we discovered that the average daily PM2.5 concentration over a 16-month period exceeded China's annual ambient air quality standard by nearly fourfold. This is the first report exploring the effects of long-term PM2.5 on hormones secreted by the HPA, HPT, and HPG axes, and their subsequent interplay with inflammatory markers in both sexes of rats. Our findings offer new perspectives on irregular endocrine system function.

We observed that PM2.5 exposure curtailed weight gain in male and female rats, suggesting it jeopardizes rat health particularly during aging. Similar to our previous study, we found that FA reduced the body weight with only in male rat after four months of exposure [6] and lasted for one year after PM2.5 exposure. Interesting, we also found that FA reduced the body weight in female rat after one year exposure. During the 16-month exposure, PM2.5 levels surpassed national air quality thresholds, pointing out potential metabolic disturbances that potentially result in adverse events. These data accentuate that aging exacerbated the deleterious health impact imposed by PM2.5 exposure, highlighting the urgency for establishing effective PM2.5 reduction strategies, especially important for elder population.

Previous studies have suggested that PM2.5 rendered immune system more reactive and induced peripheral systemic inflammation and neuroinflammation [29, 30]. PM2.5 also induced systemic inflammation and oxidative stress in rats with intracranial atherosclerosis [29]. In PM2.5-polluted human brain modelling system, PM2.5 particles penetrated blood-brain barrier (BBB) and initiated astrogliosis, resulting in slight neuronal loss and microglial infiltration [30]. Our investigation delineated a noteworthy link between chronic PM2.5 exposure and elevated inflammatory markers during aging. For example, we found that long-term PM2.5 exposure increased IL-6 and TNF-*α* levels in male and female rats. Our qRT-PCR result also indicated that PM2.5 differentially increased proinflammatory cytokines IL-6 and TNF-*α* in cortex, hippocampus, and hypothalamus of male and female rats. Previous studies have suggested the link between PM2.5 exposure and nervous system diseases [31, 32]. Thus, chronic PM2.5 exposure-induced neuroinflammation may worsen the outcomes of nervous system diseases such as stroke and Alzheimer's disease during aging. However, epidemiological and toxicological studies from other groups showed that the effects of long-term PM2.5 exposure was limited, which was contrast to the short-term exposure as reported [33].

Our results also suggested the link between chronic PM2.5 exposure and endocrine imbalances during aging. Crosstalk among the endocrine axes including HPA, HPG, or HPT does not act independently of each other, which may exceed the border of one axis itself. Concentrated ambient PM2.5 exposure has been found to be involved in hypothalamic inflammation in a NF-*κ*B signaling dependent manner [8, 13, 34]. Prior studies have delved into PM2.5's effect on hormone levels from the HPA, HPT, and HPG axes [6, 11, 35–37]. The HPA axis is responsible for releasing adrenal corticosteroids cortisol or corticosterone in response to stress. Most previous studies found that short-term PM2.5 exposure increased the HPA activity with high hormone levels. Unlike these results, we shed new light on the health risks associated with prolonged PM2.5 levels, particularly in the elderly residing in heavily polluted areas. From our results, we see that the levels of CRH, ACTH, and CORT of HPA axis almost decreased after long-term PM2.5 exposure. Our study may underscore the importance of early intervention to mitigate PM2.5-induced endocrine disruptions.

HPT axis determines the biochemical processes by producing thyroid hormones, including T4 and T3. Earlier studies have indicated that the HPT axis was vulnerable to environmental contaminants [38–40]. There was also an association between prenatal exposure to air pollutants and newborn T4 levels [41]. Air pollution exposure may influence other thyroid function parameters, such as free thyroxine (FT4) and free triiodothyronine (FT3) [42]. We found that extended PM2.5 exposure led to a decline in TRH and T3 although it was more pronounced in lowering TSH in females. However, no notable sex difference in T4 levels was observed. These results are consistent with our previous study in which HPT axis activity was decreased after PM2.5 exposure for six months. These results point out the possible thyroid malfunction and emphasize the likelihood that prolonged PM2.5 exposure may disrupt thyroid regulation leading to adverse health events.

HPG axis modulates reproductive functions and fertility by production GnRH, FSH, LH, and sex steroid hormones. HPG axis is instrumental for fertility. Existing research hints at reduced reproductive function with elevated PM2.5 levels [12, 43–45]. PM2.5 exposure-induced hypothalamic inflammation suppressed HPG axis and subsequently impaired spermatogenesis [23]. PM2.5 may potentially promote negative outcomes during pregnancy and male and female fertility [46]. Consistent with previous studies, long-term PM2.5 exposure led to diminished HPG axis hormones, possibly affecting reproductive capacity in male and female rats. Although fertility is not a concern for the elderly, such hormonal shifts may reduce the quality of life or trigger other ailments [47, 48]. Our findings highlight the risk for long-term PM2.5 interaction with aging in disrupting HPG axis stability, potentially resulting in adverse reproductive health outcomes.

The current study enhances our comprehension of long-term health implications after PM2.5 exposure in several ways. First, it considers a prolonged 16-month exposure duration, which is longer than many previous studies. Furthermore, our study used fluctuating daily PM2.5 concentrations, reflecting true exposure scenarios. However, several limitations merit consideration. First, we did not assess the morphological changes in HPA-, HPT-, and HPG-related organs post PM2.5 exposure. Second, we did not evaluate the expression of all genes along these axes, limiting our insights into the molecular mechanics underlying hormonal changes. Lastly, hormone levels at different exposure intervals were not examined, restricting our understanding of the time-dependent nature of these effects.

In summary, our findings suggest that chronic PM2.5 exposure may stimulate inflammation and alter hormone levels produced by the HPA, HPT, and HPG axes. The possible mechanisms are that the nanoparticles can infiltrate the bloodstream to increase systemic inflammation response and reach other organs, such as brain to induce neuroinflammation. Then, HPA, HPT, and HPG axes are active to release hormone. Our study provides valuable hints for reducing neuroinflammation and managing endocrine system abnormalities induced by long-term PM2.5 inhalation to avoid nervous system diseases, such as stroke and Alzheimer's disease during aging. Nonetheless, more studies are needed to elucidate the molecular dynamics and cross-axis interactions in response to PM2.5 exposure.

## Figures and Tables

**Table 1:** Primers for real-time polymerase chain reaction (PCR) analysis.

Genes	Forward primer (5′–3′)	Reverse primer (5′–3′)
IL-6	GATTGTATGAACAGCGATGATGC	AGAAACGGAACTCCAGAAGACC
IL-1*β*	CCCAACTGGTACATCAGCACCTCTC	CTATGTCCCGACCATTGCTG
TNF-*α*	TGAACTTCGGGGTGATCGGT	GGCTACGGGCTTGTCACTCG
GAPDH	TTCCTACCCCCAATGTATCCG	CCACCCTGTTGCTGTAGCCATA

## Data Availability

The data used to support the findings of this study are included within the article.
